# *N*-glycosylation of Rim21 at an Unconventional Site Fine-tunes Its Behavior in the Plasma Membrane

**DOI:** 10.1247/csf.19021

**Published:** 2019-11-30

**Authors:** Keisuke Obara, Tetsuya Kotani, Hitoshi Nakatogawa, Akio Kihara, Takumi Kamura

**Affiliations:** 1 Division of Biological Science, Graduate School of Science, Nagoya University, Furo-cho, Chikusa-ku, Nagoya 464-8602, Japan; 2 School of Life Science and Technology, Tokyo Institute of Technology, Nagatsuta-cho 4259 B-20, Midori-ku, Yokohama 226-8501, Japan; 3 Faculty of Pharmaceutical Sciences, Hokkaido University, Kita 12-jo Nishi 6-chome, Kita-ku, Sapporo 060-0812, Japan

**Keywords:** plasma membrane, lipid asymmetry, *N*-linked glycosylation, microdomain, *Saccharomyces cerevisiae*

## Abstract

The polytopic plasma membrane protein Rim21 senses both the elevation of ambient pH and alterations in plasma membrane lipid asymmetry in the Rim101 pathway in budding yeast. Rim21 is known to undergo *N*-glycosylation, but the site and function of the glycosylation modification is not known. Using a systematic mutation analysis, we found that Rim21 is *N*-glycosylated at an unconventional motif located in the N-terminal extracellular region. The Rim21 mutant protein that failed to receive *N*-glycosylation showed prolonged protein lifetime compared to that of WT Rim21 protein. Although both the WT and mutant Rim21 localized to the plasma membrane, they exhibited different biochemical fractionation profiles. The mutant Rim21, but not WT Rim21, was mainly fractionated into the heavy membrane fraction. Further, compared to WT Rim21, mutant Rim21 was more easily solubilized with digitonin but was conversely more resistant to solubilization with Triton X-100. Despite these different biochemical properties from WT Rim21, mutant Rim21 protein could still activate the Rim101 pathway in response to external alkalization. Collectively, *N*-glycosylation of Rim21 is not indispensable for its activity as a sensor protein, but modulates the residence of Rim21 protein to some microdomains within the plasma membrane with distinct lipid conditions, thereby affecting its turnover.

## Introduction

Living organisms always need to sense the extracellular environment and respond to its alterations. Microorganisms that can grow over a wide range of pH, *e.g.* the budding yeast *Saccharomyces cerevisiae* and a filamentous fungi *Aspergillus nidulans*, activate the Rim101 pathway to cope with external alkalization stress (Davis, 2009; Peñalva and Arst, 2002; Selvig and Alspaugh, 2011). Since this signaling pathway is required for the proliferation and virulence of pathogenic fungi such as *Cryptococcus neoformans* and *Candida albicans* in host organisms, understanding the Rim101 pathway is important for medical and agricultural applications as well as for basic biology.

In the lipid bilayer of the plasma membrane in eukaryotes, lipid molecules are distributed unevenly between the two leaflets, forming an asymmetric membrane (Devaux, 1991; Verkleij and Post, 2000). For instance, phosphatidylserine and phosphatidylethanolamine are mostly confined to the inner (cytosolic) leaflet whereas phosphatidylcholine and sphingolipids are enriched in the outer (extracellular) leaflets. Maintenance and proper regulation of this lipid asymmetry is essential for cell viability (Hua *et al.*, 2002), and in humans, mutations in genes encoding proteins that catalyze transbilayer movements of lipid molecules are implicated in diseases including cholestasis, Stargardt macular dystrophy, and Scott syndrome (Allikmets *et al.*, 1997; Bull *et al.*, 1998; Toti *et al.*, 1996). In yeast, the Rim101 pathway senses alterations in plasma membrane lipid asymmetry in addition to ambient alkalization and invokes adaptation responses (Ikeda *et al.*, 2008; Kihara and Igarashi, 2004; Makuta *et al.*, 2017; Yamauchi *et al.*, 2015). Although how the Rim101 pathway can mediate distinct signals of external alkalization and changes in lipid asymmetry is yet to be defined, it is proposed that external alkalization alters the lipid asymmetry, which in turn is sensed by the Rim101 pathway (Nishino *et al.*, 2015; Obara *et al.*, 2012).

In the Rim101 pathway, Rim21, a polytopic plasma membrane protein, acts as a sensor protein (Obara *et al.*, 2012). Rim21 forms a sensor complex with other plasma membrane proteins Dfg16 and Rim9, which is required for the stability and plasma membrane localization of Rim21 (Obara *et al.*, 2012). The C-terminal cytosolic region of Rim21 (Rim21C) contains the sensor motif for altered lipid asymmetry (Nishino *et al.*, 2015). Rim21C is usually associated with the plasma membrane; however, upon external alkalization or altered lipid asymmetry, it dissociates from the plasma membrane. Activated Rim21 recruits an arrestin-like protein Rim8, the ESCRT complex, Bro1-like protein Rim20, and a calpain-like protease Rim13 that proteolytically activates Rim101 to the plasma membrane (Galindo *et al.*, 2012; Herrador *et al.*, 2010; Obara and Kihara, 2014). Rim21 has ideal characteristics for a potential drug target against fungal infection. First, it regulates the Rim101 pathway that is required for the virulence of fungal pathogens at the most upstream position. Second, it is exposed to the cell surface, which allows easier access to chemical compounds. Finally, no gene possessing a significant homology with fungal *RIM21* can be found in the genomes of human and agricultural crops, though functional homologs without sequence similarity might exist, which lowers the risk of side-effects from the developed drugs. Therefore, deeper understanding of Rim21 may enable novel approaches to prevent animal and plant fungal infections.

Rim21 is a polytopic membrane protein presumably spanning the membrane seven times with its N- and C-terminus facing the extracellular space and cytosol, respectively (Obara *et al.*, 2012). Comprehensive proteomic analyses have shown that Rim21 undergoes phosphorylation at least two serine residues (Albuquerque *et al.*, 2008; Bodenmiller *et al.*, 2008). Phosphorylation at the two known sites is suggested to be dispensable for Rim21 function (Nishino *et al.*, 2015). Rim21 is known to undergo *N*-glycosylation as well (Obara *et al.*, 2012). The site and role of *N*-glycosylation in Rim21 is not known. In this study, we investigated the *N*-glycosylation of Rim21 in detail. We found that Rim21 undergoes *N*-glycosylation at an unconventional motif. *N*-glycosylation of Rim21 at this site is required for proper localization of Rim21 to the lipid microenvironment within the plasma membrane thus fine-tuning the Rim21 behavior.

## Materials and Methods

### Yeast strains and media

The *Saccharomyces cerevisiae* strains used in this study are listed in Table I. Yeast cells were grown in synthetic medium (2% D-glucose and 0.67% yeast nitrogen base without amino acids with appropriate supplements) at 30°C. For alkaline treatment to activate the Rim101 pathway, Tris-HCl (pH 8.0) was added to the culture medium at a final concentration of 100 mM.

### Genetic manipulation

The *RIM21-N15Q-2GFP* strain was constructed as follows. The *N15Q* mutation was introduced into pOK692 (*RIM21-2GFP*) (Nishino *et al.*, 2015) using the QuikChange site-directed mutagenesis kit (Agilent Technologies, Santa Clara, CA), creating pOK793. We then linearized pOK793 *via* digestion with *Fsp*AI and inserted it into the *RIM21* locus of YOK2027 (*rim21*Δ) cells. The *mRFP-SNC1(pm)* sequence was introduced as follows. The pKT2165 [pRS305-*P_TPI1_*-*mRFP-SNC1(pm)*] plasmid [kindly provided by Dr. K. Tanaka (Hokkaido University, Japan) (Takeda *et al.*, 2014)] was linearized by digestion with *Eco*RV and inserted into the *LEU2* locus. *RIM21-N15Q-HA* and *RIM21-N15Q* plasmids were constructed by site-directed mutagenesis using the QuikChange site-directed mutagenesis kit (Agilent Technologies). *RIM21-HA* plasmid (pOK313) (Obara *et al.*, 2012) and *RIM21* plasmid (pOK648) (Nishino *et al.*, 2015) were used as templates. Successful construction of the plasmids was confirmed by DNA sequencing. The pFI1 plasmid encoding *HA-RIM101* was provided by Dr. T. Maeda (Hamamatsu University School of Medicine, Japan).

### Deglycosylation

Proteins in total lysates were denatured at 37°C for 10 min in SDS sample buffer, and then diluted with a 4×volume of endoglycosidase H (Endo H) buffer [62.5 mM sodium citrate (pH 5.5) and 1.25 mM PMSF]. Endo H (Endo H_f_; New England Biolabs, Beverly, MA) was added to a final concentration of 20 U/μL. The mixture was incubated at 37°C for 1 h, treated with an appropriate volume of 4×SDS sample buffer at 37°C for 10 min, and subjected to immunoblot analysis.

### Immunoblot analysis

Total cell lysates were prepared by the alkaline/trichloroacetic acid method and subjected to immunoblot analyses as described previously (Obara *et al.*, 2006). Anti-HA TANA2 (0.5 μg/ml; Medical & Biological Laboratories, Nagoya, Japan), anti-Pgk1 (0.5 μg/ml; Thermo Fisher Scientific, Waltham, USA), anti-DDDDK-tag FLA-1 (0.5 μg/ml; Medical & Biological Laboratories), anti-Pma1 yN-20 (0.1 μg/ml; Santa Cruz Biotechnology, Dallas, USA), anti-Pep12 2C3G4 (0.5 μg/ml; Thermo Fisher Scientific, Waltham, USA), and anti-Sec12 (provided by Dr. A. Nakano, RIKEN) antibodies were used as the primary antibodies, and HRP-conjugated anti-mouse IgG F(ab’)_2_ (1:7,500 dilution; GE Healthcare Life Sciences, Little Chalfont, UK), HRP-conjugated anti-mouse IgA F(ab’)_2_ (1:7,500 dilution; Thermo Fisher Scientific) and HRP-conjugated anti-goat IgG F(ab’)_2_ (1:7,500 dilution; Santa Cruz Biotechnology) fragments were used as the secondary antibodies. Immunodetection was performed using a Luminata Forte Western HRP Substrate system (Merck Millipore, Burlington, MA) with a bioanalyzer (LAS4000 mini; GE healthcare Biosciences, Piscataway, NJ) or with X-ray films.

### Co-immunoprecipitation

Cells were cultured in synthetic medium lacking His to the log phase, harvested, suspended in lysis buffer [50 mM Tris-HCl (pH 7.5), 150 mM NaCl, 1 mM dithiothreitol, 1 mM PMSF, and EDTA-free protease inhibitor cocktail (Complete; Roche Diagnostics, Indianapolis, IN)], and broken by mixing vigorously with glass beads at 4°C for 10 min. The cell lysates were sonicated and centrifuged at 3,000×*g* for 3 min to remove cell debris. After treatment with 1% Triton X-100 at 4°C for 30 min, samples were ultracentrifuged at 100,000×*g* for 30 min. The supernatant was then incubated with anti-HA 3F10 (Roche Diagnostics) and HM-Protein G magnetic beads (Tamagawa Seiki, Iida, Japan) while rotating, and was maintained at 4°C for 90 min. The beads were collected magnetically and washed two times with lysis buffer containing 1% Triton X-100. The bound proteins were eluted with SDS sample buffer.

### Cycloheximide chase

Cells were cultured in synthetic medium lacking His and Leu to the log phase and treated with or without alkali (100 mM Tris-﻿HCl, pH 8.0) for 20 min. A portion of the cell suspension was harvested as the “Time 0” sample. Cells were then treated with 200 μg/ml cycloheximide (Nacalai Tesque, Kyoto, Japan), harvested at 30 and 60 min, and subjected to immunoblot analysis.

### Microscopy

Cells were grown to the log phase in synthetic medium and subjected to microscopic observation before and after the alkaline treatment. Fluorescence of GFP and mRFP were visualized under a fluorescence microscope (IX-83, Olympus, Tokyo, Japan) equipped with an electron-multiplying CCD camera (ImagEM, C9100-13, Hamamatsu Photonics, Hamamatsu, Japan) and a 150× objective lens (UAPON 150XOTIRF, NA/1.45, Olympus) as reported previously (Kotani *et al.*, 2018). GFP and mRFP were excited with a 488-nm blue laser (50 mW; Coherent, Santa Clara, CA) and a 588-nm yellow laser (50 mW; Coherent), respectively. Fluorescence was filtered with a dichroic mirror reflecting 405-, 488-, and 588-nm wavelengths (Olympus) and separated into two channels using the DV2 multichannel imaging system (Photometrics, Tucson, AZ) equipped with a Di02-R594-25x36 dichroic mirror (Semrock, Rochester, NY). GFP and mRFP fluorescence was further filtered with a TRF59001-EM ET bandpass filter (Chroma Technology, Bellows Falls, VT) and an FF01-624/40-25 bandpass filter (Semrock), respectively. Images were acquired using MetaMorph software (Molecular Devices, Sunnyvale, CA) and processed using Photoshop CS3 (Adobe, San Jose, CA).

## Results

### Rim21 undergoes N-glycosylation at an unconventional motif

**Fig. 1:**
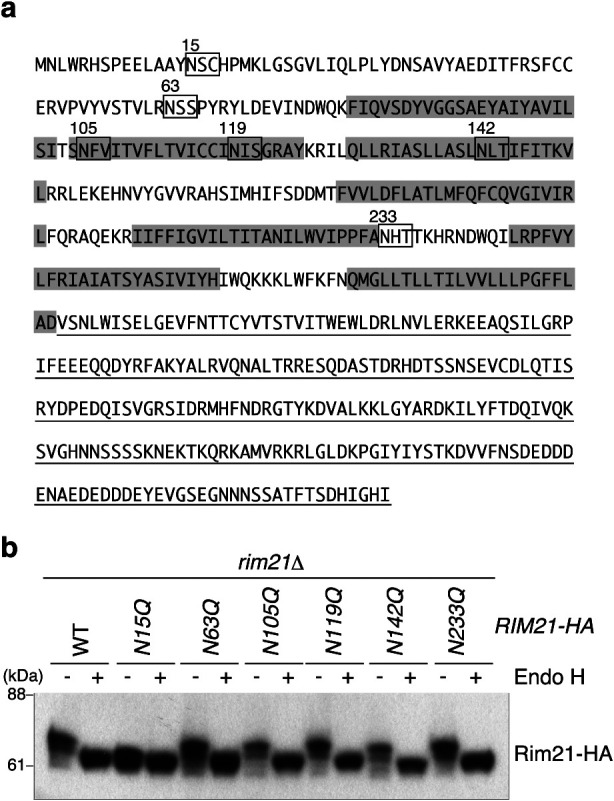
Rim21 receives *N*-glycosylation at N15 residue. (a) Amino acid sequence of Rim21. The C-terminal cytosolic region is underlined. Sequences that match the potential conventional and unconventional *N*-glycosylation motifs are boxed. Predicted transmembrane segments are highlighted. (b) YOK2027 (*rim21*Δ) cells harboring an expression vector for WT or the mutant Rim21-HA were grown to the log phase, and their total lysates were prepared. The total lysates were then treated with or without Endo H and subjected to immunoblot analysis with the anti-HA antibody.

In order to determine the *N*-glycosylation site of Rim21, we performed a systematic mutation analysis of Rim21. The *N*-glycan chain is added to Asn residues in the consensus sequence NXS/T (where X signifies any amino acid except proline). In addition to this consensus motif, recent glycoproteomic analysis revealed unconventional, but very rare, *N*-glycosylation sites including NXC, NGX, and NXV (Zielinska *et al.*, 2010). We substituted the Asn residues of potential conventional and unconventional *N*-glycosylation motifs with Gln (Fig. 1a). Since Rim21C is located in the cytosol (Obara *et al.*, 2012), where *N*-glycosylation does not take place, Gln substitution in this region was omitted. *N*-glycosylation of Rim21 was detected by downshifting of the Rim21-HA band in immunoblot analysis using lysates treated with or without endoglycosidase H (Endo H) which cleaves the *N*-linked glycan chains. In mammalian cells, *N*-glycan chains attached to Golgi and post-Golgi proteins are resistant to Endo H treatment because of fucosylation with the first *N*-acetylglucosamine. However, this fucosylation does not occur in yeast cells; therefore *N*-glycan chains attached to Golgi- and post-Golgi proteins are cleaved by Endo H like those attached to ER proteins. Unmutated or mutated Rim21-HA variants were expressed in *rim21*Δ cells from a single-copy (*CEN6*) plasmid under the control of its own promoter, and their *N*-glycosylation was analyzed. Gln substitution at the conventional NXS/T motifs (N63Q, N119Q, N142Q, or N233Q) did not affect the mobility of Rim21-HA (Fig. 1b). Endo H treatment of these lysates caused downshifting of the Rim21-HA bands, indicating that these mutant Rim21-HA proteins still underwent *N*-glycosylation. In contrast, N15Q mutation at the unconventional NSC motif caused downshift of the Rim21-HA band in SDS-PAGE even without Endo H treatment. Endo H treatment did not affect the mobility of the N15Q mutant protein. These results indicate that Rim21 undergoes *N*-glycosylation at the N15 residue, and that this is the sole *N*-glycosylation site in Rim21.

### Rim21 without N-glycosylation retains sensor activity

**Fig. 2:**
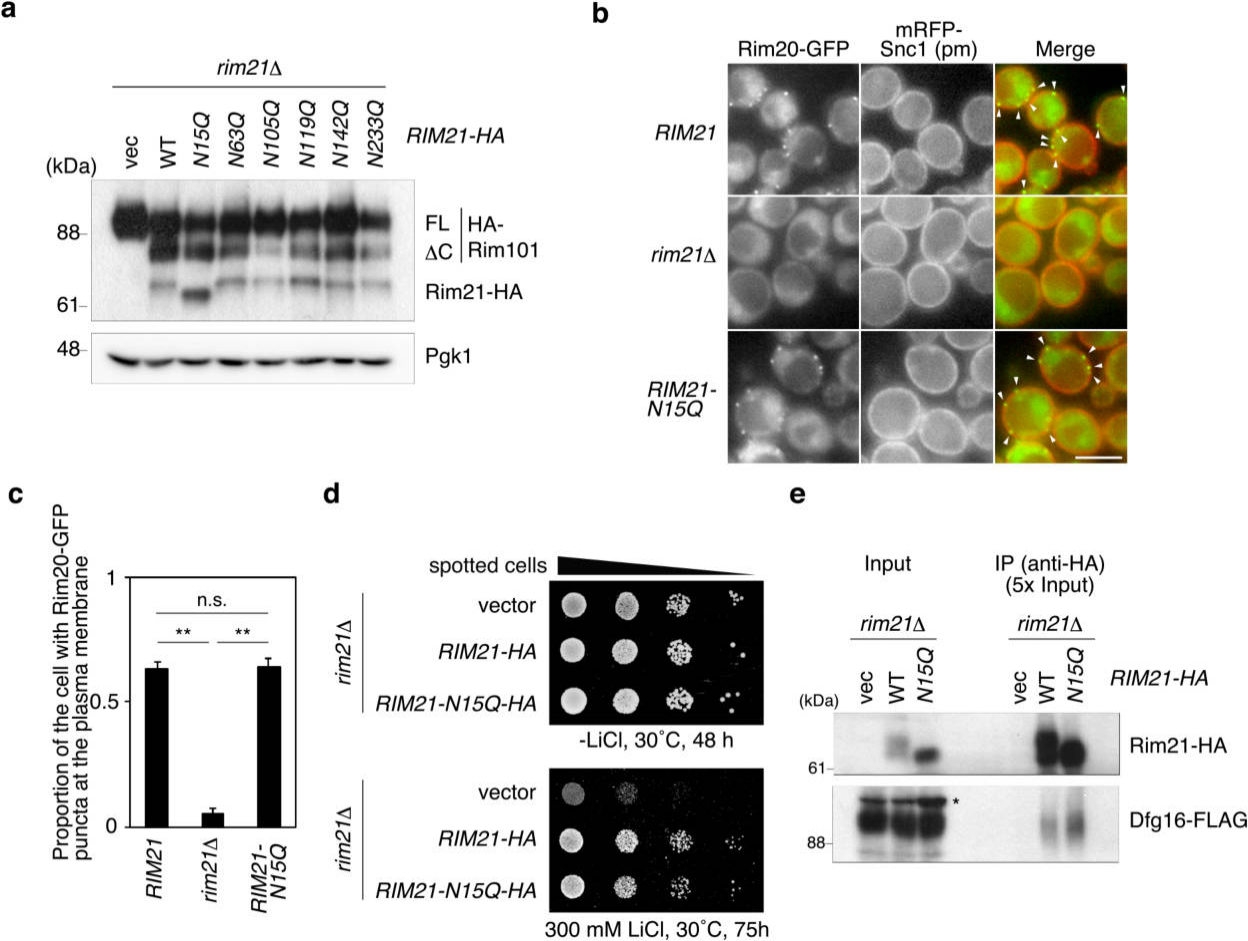
Rim21-N15Q mutant protein is functional. (a) YOK2027 (*rim21*Δ) cells expressing HA-Rim101 and one of the HA-tagged Rim21 mutants were grown to the log phase and harvested at 30 min of alkaline-treatment. Their total lysates were prepared and subjected to immunoblot analysis using anti-HA and anti-Pgk1 antibodies. (b) YOK5376 [*RIM20-GFP mRFP-SNC1(pm)*], YOK5377 [*rim21*Δ* RIM20-GFP mRFP-SNC1(pm)*], and YOK5378 [*rim21*Δ* RIM20-GFP mRFP-SNC1(pm) RIM21-N15Q*] cells were grown to log phase, alkaline-treated for 90 min, and observed under a fluorescence microscope. Bar, 5 μm. Arrowheads indicate Rim20 complex at the plasma membrane. (c) Quantification of cells exhibiting Rim20-GFP puncta at the plasma membrane. Values represent the mean ± SD of three independent cultures (>100 cells counted per culture). **, *P* < 0.01; n.s., not significant. (d) Cells were grown to a stationary phase in synthetic medium lacking His (SD-His), serially diluted at 1:10, spotted on -His plates with or without 300 mM LiCl, and grown at 30°C for the indicated periods. (e) Total lysates were prepared from YOK3632 (*rim21*Δ *DFG16-FLAG*) cells harboring an empty vector or either of the expression vectors for WT or N15Q mutant Rim21, and their membrane fractions were collected by ultracentrifugation. The membrane fractions were solubilized with Triton X-100, and immunoprecipitated using anti-HA antibody. Immunoprecipitates were separated by SDS-PAGE and detected by immunoblotting with anti-HA and anti-FLAG antibodies. Asterisk indicates nonspecific bands. IP: immunoprecipitation.

We next investigated the function of Rim21 *N*-glycosylation. Rim21 is located at the most upstream position of the Rim101 pathway. At the final step of the Rim101 pathway, the transcription factor Rim101 undergoes proteolytic activation (Galindo *et al.*, 2012; Herrador *et al.*, 2010; Obara and Kihara, 2014; Peñalva and Arst, 2002), which can be monitored by immunoblotting for Rim101. Rim21 was activated by external alkalization, and the processing of Rim101 was monitored. Rim101 was normally processed in response to external alkalization in *rim21*Δ cells expressing the N15Q mutant protein from a single-copy (*CEN6*) plasmid under the control of its own promoter (Fig. 2a), indicating that the Rim21-N15Q mutant protein is capable of stimulating the Rim101 pathway. The function of Rim21 can also be detected by observing the Rim20 complex recruited to the plasma membrane upon Rim21 activation. Rim20-GFP puncta at the plasma membrane, visualized by an established plasma-membrane marker [mRFP-Snc1(pm) (Takeda *et al.*, 2014)], were detected in *rim21*Δ cells expressing Rim21 after alkaline treatment (Fig. 2b and c). Although intracellular Rim20-GFP dots were also occasionally observed as background, a previous study noted that the Rim101 signaling complex, including Rim20, at the plasma membrane but not at intracellular compartments is involved in alkaline signal transduction (Obara and Kihara, 2014). Likewise, Rim20-GFP assembled at the plasma membrane in *rim21*Δ cells expressing the Rim21-N15Q mutant protein, indicative of normal activity of the Rim21-N15Q mutant. Mutants in the Rim101 pathway are known to be hypersensitive to LiCl (Hayashi *et al.*, 2005). Expression of the Rim21-N15Q mutant from a single-copy (*CEN6*) plasmid under the control of its own promoter in *rim21*Δ cells suppressed the sensitivity to LiCl similar to WT Rim21 (Fig. 2d), further supporting that Rim21-N15Q protein retains its activity to invoke the Rim101 pathway. Rim21 forms a protein complex with Dfg16, which is essential for the stability and plasma membrane-localization of Rim21 (Obara *et al.*, 2012). Both WT and Rim21-N15Q co-immunoprecipitated Dfg16 (Fig. 2e). All these results indicate that *N*-glycosylation of Rim21 is not a prerequisite for Rim21 function.

### N-glycosylation of Rim21 prolongs the lifetime of Rim21

**Fig. 3:**
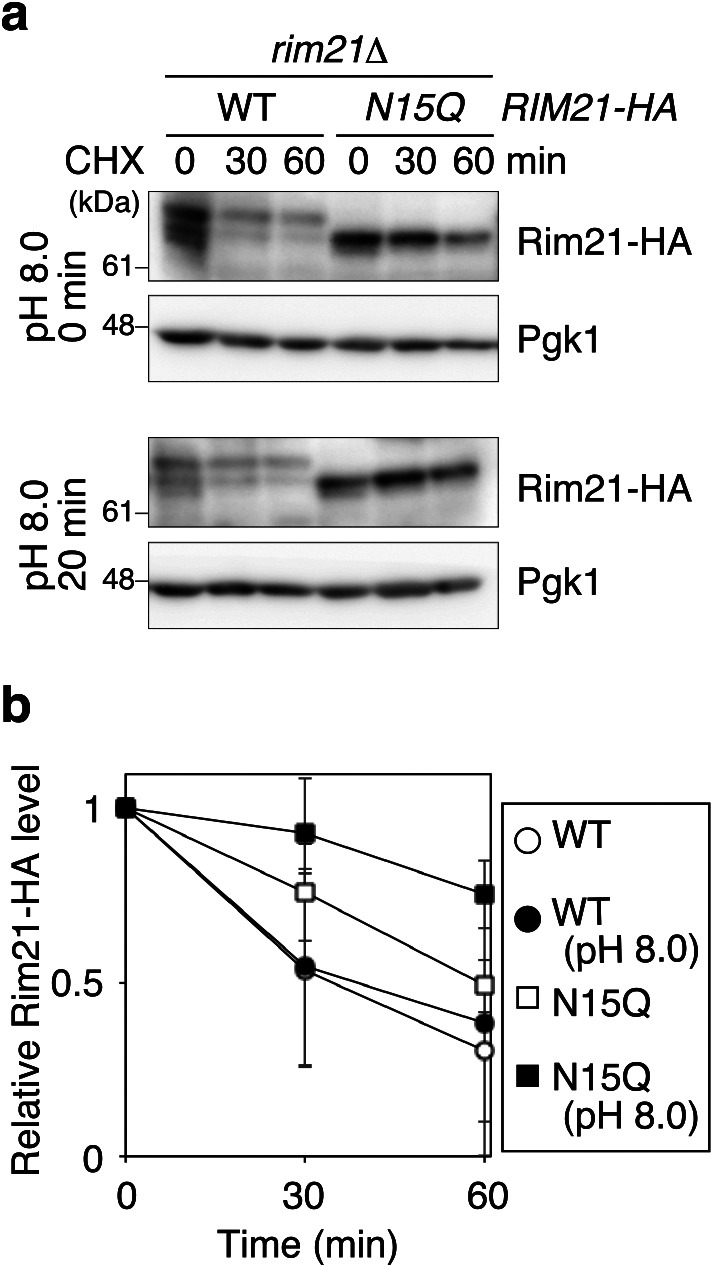
Rim21-N15Q is turned over more slowly than WT Rim21. (a) YOK2027 (*rim21*Δ) cells expressing Rim21-HA or Rim21-N15Q-HA were grown to the log phase and mock- or alkali-treated for 20 min. Cells were then treated with 200 μg/ml cycloheximide for the indicated periods and harvested. Their total lysates were subjected to immunoblot analysis with anti-HA and anti-Pgk1 antibodies. (b) Rim21-HA and Rim21-N15Q-HA levels were measured, normalized with those of Pgk1, and shown as a relative value to those at 0 min. Values represent the mean ± SD from three independent experiments.

During the investigation of the function of Rim21 *N*-glycosylation, we noticed that the cellular level of Rim21-N15Q is higher than that of WT Rim21 (Fig. 2a). We thus assumed that the Rim21-N15Q mutant is more stable than WT Rim21, which could be examined by a cycloheximide chase experiment. As cycloheximide inhibits *de novo* protein synthesis, we can evaluate the lifetime of each protein using this drug. As shown in Fig. 3, the lifetime of Rim21-N15Q was longer than that of WT Rim21, indicating that the turnover of Rim21-N15Q proceeds more slowly than that of WT Rim21. This was also the case for alkali-treated cells.

### N-glycosylation of Rim21 modulates its residence to the plasma membrane lipid microdomains

**Fig. 4:**
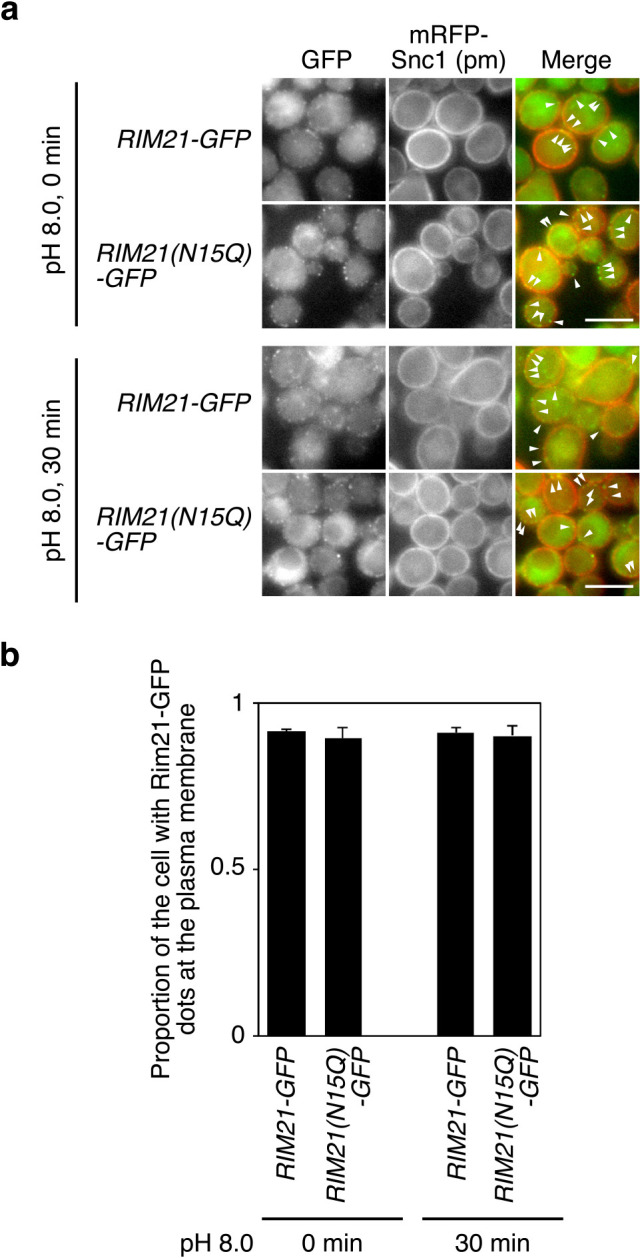
Rim21-N15Q localizes to the plasma membrane. (a) YOK5374 [*RIM21-2GFP mRFP-SNC1(pm)*] and YOK5375 [*RIM21-N15Q-2GFP* *mRFP-SNC1(pm)*] cells were grown to log phase in synthetic medium and subjected to fluorescence microscopy before and 30 min after alkaline treatment. The arrowhead indicates the GFP signal merged with the mRFP-Snc1(pm) signal. Bar, 5 μm. (b) Quantification of cells exhibiting GFP signals primarily at the plasma membrane. Values represent the mean ± SD of three independent cultures (>100 cells counted per culture).

**Fig. 5:**
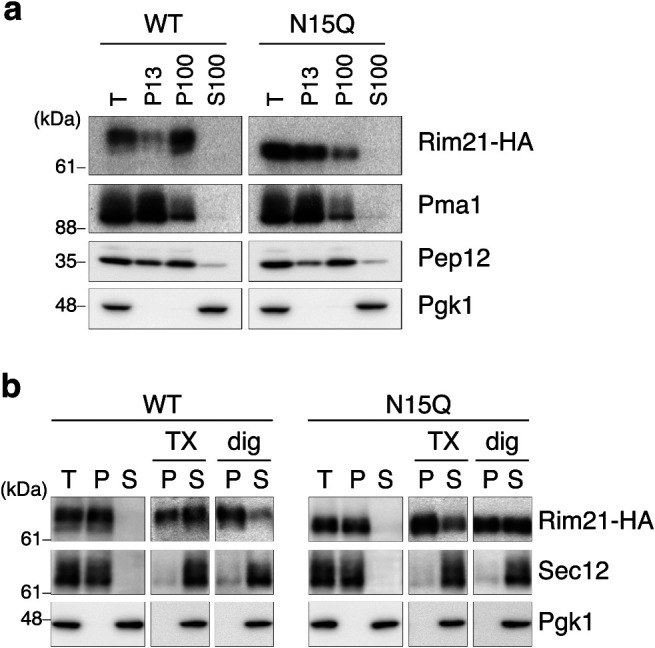
WT Rim21 and Rim21-N15Q localize to different lipid microenvironments. (a) YOK2027 (*rim21*Δ) cells expressing Rim21-HA or Rim21-N15Q-HA were grown to the log phase and their total lysates were subjected to a sequential fractionation assay. P13 and P100, are the sedimented fractions of centrifugation at 13,000×*g* and 100,000×*g*, respectively. S100, is the supernatant fraction of centrifugation at 100,000×*g*. (b) YOK2027 (*rim21*Δ) cells expressing Rim21-HA or Rim21-N15Q-HA were grown to the log phase and their total lysates were subjected to ultracentrifugation before or after solubilization with 1% Triton X-100 (TX) or 1% digitonin (dig). Sec12 and Pgk1 are markers for transmembrane protein and soluble protein, respectively. T, total; P, pellet; S, supernatant.

Given the prolonged protein lifetime of the Rim21-N15Q mutant, we hypothesized that Rim21-N15Q localizes, at least in part, to a different microenvironment in the plasma membrane compared to WT Rim21. We confirmed that Rim21-N15Q-GFP localizes mainly to the plasma membrane and in some portion to the intracellular punctate structure observed for WT Rim21-GFP before and after alkaline-treatment (Fig. 4). Next, we investigated the biochemical microenvironment where Rim21-N15Q mutant protein localizes. Cells were lysed and fractionated by stepwise centrifugation. WT Rim21-HA was fractionated into P100 (light membrane) and, to a lesser extent, in P13 (heavy membrane) fractions (Fig. 5a). In contrast, Rim21-N15Q mutant was fractionated mainly into the P13 fraction and to a minor extent into the P100 fraction. This different fractionation suggests that the WT Rim21 and Rim21-N15Q mutant localize to distinct microenvironments in the plasma membrane. This notion was further examined by comparing the solubility of the WT and mutant Rim21 in detergents. WT Rim21 was more solubilized by 1% Triton X-100 compared to the Rim21-N15Q mutant protein (Fig. 5b). In contrast, Rim21-N15Q was more efficiently solubilized by 1% digitonin compared to WT Rim21. Both detergents efficiently solubilized the single pass membrane protein Sec12, indicating successful solubilization and fractionation. Collectively, the results suggest that Rim21-N15Q resides, at least partly, in a distinct local environment within the plasma membrane compared to WT Rim21.

## Discussion

In this research, we found that Rim21 undergoes *N*-glycosylation at an unconventional motif (Fig. 1). This unconventional *N*-glycosylation was found to be dispensable for activating the Rim101 pathway (Fig. 2). However, in this research, cells were grown under nutrient-rich and unstressed conditions; therefore, we cannot exclude the possibility that Rim21 *N*-glycosylation might be important under some stressed conditions. Loss of *N*-glycosylation in Rim21 affected the behavior of Rim21 (Fig. 3 and Fig. 5). Biochemical characterization of the Rim21-N15Q mutant suggested that this mutant protein localizes to a different microenvironment in the plasma membrane compared to the region of WT Rim21 localization. Notably, the Rim21-N15Q mutant was more solubilized by digitonin compared to WT Rim21. Digitonin is known to have high affinity to sterols, and is often used to solubilize sterol-rich raft-like membranes (Elias *et al.*, 1978). One possibility is that the Rim21-N15Q mutant has higher affinity to sterol-rich microdomains compared to WT Rim21. Yeast plasma membrane contains at least three distinct subdomains; 1) MCC (membrane compartment occupied by Can1), a sterol-rich stable region where the membrane protein Can1 is enriched, 2) MCP (membrane compartment occupied by Pma1), a subdomain defined as the plasma membrane region containing readily diffusible proteins including the plasma membrane ATPase Pma1, and 3) movable membrane subdomain that houses TORC2 (Douglas *et al.*, 2011; Malinsky *et al.*, 2010). Importantly, proteins of the endocytic machinery are excluded from MCC, and thus MCC represents a protective area to control endocytosis-mediated turnover of membrane proteins (Grossmann *et al.*, 2008). It is possible that the Rim21-N15Q mutant has higher affinity to sterol-rich MCC compared to WT Rim21 and may escape from endocytosis-mediated turnover, leading to a prolonged protein lifetime. A previous study reported that endocytosis is dispensable for the Rim101 pathway (Obara and Kihara, 2014); therefore, it is consistent that cells expressing the Rim21-N15Q mutant protein can normally activate the Rim101 pathway (Fig. 2).

Recent studies have highlighted the novel function of carbohydrate chains exposed to the extracellular space, in that, interaction of the carbohydrate moiety of GPI-anchored proteins facilitates the formation of a dimer. It is proposed that this dimer is the minimum unit of the specific membrane microenvironment and serves as a platform to generate larger membrane subdomains (Suzuki *et al.*, 2017, 2018). Therefore, it is possible that the carbohydrate moiety of plasma membrane proteins laterally directs the proteins to specific membrane microenvironments as well as regulates formation of the microenvironment itself. In a similar context, interaction between the *N*-glycan chains attached to Rim21 and/or neighboring proteins might fine-tune the localization of Rim21 to specific microenvironments in the plasma membrane and thereby modulate its dynamics. Detailed monitoring of the behavior of Rim21 and the N15Q mutant in future may provide a novel paradigm of how *N*-glycan chains influence the localization of membrane proteins and formation of the lipid microenvironment.

## Acknowledgments

We would like to thank Dr. T. Maeda (Hamamatsu University School of Medicine) and Dr. K. Tanaka (Hokkaido University) for providing the *HA-RIM101* plasmid (pFI1) and the *mRFP-SNC1* plasmid, respectively. We also thank Dr. A. Nakano (RIKEN) for providing the anti-Sec12 antibody. We would like to thank Editage (www.editage.com) for English language editing. This work was supported by JSPS KAKENHI Grant Numbers JP16K07288, JP18K19292, JP 19K06561. Funding was also provided by The Hori Sciences And Arts Foundation to KO.

## Conflict of interest

The authors declare that they have no conflicts of interest with the contents of this article.

## Figures and Tables

**Table I:** Yeast strains used in this study

Strain	Genotype	Source
SEY6210	MATα *his3 leu2 ura3 trp1 lys2 suc2*	(Robinson *et al.*, 1988)
YOK2027	SEY6210, *rim21*Δ*::KanMX4*	(Obara *et al.*, 2012)
YOK3632	SEY6210, *pep4*Δ*::LEU2 pbb1*Δ*::NatNT2 rim21*Δ*::TRP1 DFG16-FLAG::KanMX6*	This study
YOK5374	SEY6210, *RIM21-2GFP::KanMX4* *mRFP-SNC1(pm)::LEU2*	This study
YOK5375	SEY6210, *rim21*Δ*::KanMX RIM21(N15Q)-2GFP::URA3* *mRFP-SNC1(pm)::LEU2*	This study
YOK5376	SEY6210, *RIM20-GFP::TRP1 mRFP-SNC1(pm)::LEU2*	This study
YOK5377	SEY6210, *rim21*Δ*::KanMX RIM20-GFP::TRP1 mRFP-SNC1(pm)::LEU2*	This study
YOK5378	SEY6210, *rim21*Δ*::KanMX RIM20-GFP::TRP1 RIM21(N15Q)::URA3* *mRFP-SNC1(pm)::LEU2*	This study

## Abbreviations

Rim21C: C-terminal cytosolic domain of Rim21
SC: synthetic complete
MCC: membrane compartment occupied by Can1
MCP: membrane compartment occupied by Pma1
